# Changes in Choriocapillaris Flow Deficits Before and After the Onset of Large Choroidal Hypertransmission Defects in AMD

**DOI:** 10.1167/iovs.66.14.20

**Published:** 2025-11-12

**Authors:** Sara Beqiri, Hefu Pan, Bhagavath S. Kumar, Alessandro Berni, Mengxi Shen, Farhan E. Hiya, Yuxuan Cheng, Omar S. El-Mulki, Gissel Herrera, Omar Badla, James Kastner, Omer Trivizki, Maura Di Nicola, Robert C. O'Brien, Nadia K. Waheed, Ruikang K. Wang, Giovanni Gregori, Philip J. Rosenfeld

**Affiliations:** 1 Department of Ophthalmology, Bascom Palmer Eye Institute, University of Miami Miller School of Medicine, Miami, Florida, United States; 2 Department of Bioengineering, University of Washington, Seattle, Washington, United States; 3 Department of Ophthalmology, IRCCS San Raffaele Scientific Institute, Milan, Italy; 4 Department of Ophthalmology, Tel Aviv Medical Center, University of Tel Aviv, Tel Aviv, Israel; 5 New England Eye Center, Tufts Medical Center, Tufts University School of Medicine, Boston, Massachusetts, United States; 6 Department of Ophthalmology, University of Washington, Seattle, Washington, United States

**Keywords:** choriocapillaris, hypertransmission defect, RPE

## Abstract

**Purpose:**

Eyes with intermediate age-related macular degeneration (iAMD) underwent swept-source optical coherence tomography angiography (SS-OCTA) imaging and were evaluated longitudinally to determine if choriocapillaris flow deficits (CCFDs) developed before or after the formation of large choroidal hypertransmission defects (hyperTDs).

**Methods:**

A retrospective review was performed of prospectively collected 6 × 6-mm SS-OCTA images from eyes with iAMD that developed large hyperTDs, defined on en face images from subretinal pigment epithelium (sub-RPE) slabs positioned 64 to 400 µm beneath Bruch's membrane (BM) as bright lesions with a greatest linear dimension (GLD) ≥ 250 µm. The onset of large hyperTDs was designated as baseline T = 0; additional visits were chosen at 1 year before and at two 1-year intervals after T = 0. A grid box strategy was implemented for the analysis of CCFDs at the site where hyperTDs formed. A change in the percentage of CCFDs greater than 5% was considered to be a true change outside the repeatability limits.

**Results:**

Twenty-seven targets from 27 eyes eligible for final analysis were followed over four visits separated by 12 ± 3 months. No targets showed a marked CCFD change above 5% prior to hyperTD onset. Only two targets showed marked increases in CCFDs after hyperTD formation. A grouped analysis of all targets showed no mean CCFD change prior to hyperTD onset, but a significant change only after the onset of hyperTD.

**Conclusions:**

CCFD values did not increase prior to the onset of hyperTDs with increases in CCFDs detected after their onset. These results suggest that loss of choriocapillaris perfusion did not precede hyperTD formation but may play a role in hyperTD growth.

Age-related macular degeneration (AMD) is the leading cause of irreversible vision loss among the elderly worldwide.^1^ Geographic atrophy (GA), also known as complete retinal pigment epithelium (RPE) and outer retinal atrophy (cRORA), is the late form of nonexudative AMD and is characterized by degeneration of the photoreceptors, loss of the RPE, and the presence of choroidal hypertransmission defects (hyperTDs).^2^^–^^4^ Histopathological studies have confirmed that GA is accompanied by loss of the choriocapillaris (CC),^5^^,^^6^ the dense capillary network underlying Bruch's membrane that provides metabolic support to the RPE and outer retina.^7^^,^^8^ The RPE is also essential for maintaining the CC structure and function due to its secretion of trophic factors and its action as a physical and biochemical barrier against harmful levels of visual cycle byproducts such as 11-*cis* retinoids.^9^^–^^14^ Although the end-stage of GA unambiguously involves the degeneration of both the CC and RPE, due to their symbiotic relationship it is debated whether the loss of the CC is the initiating factor, whether the loss of the RPE initiates the loss of the CC, or whether both layers are lost synchronously as AMD progresses to its late stage.^15^

Large choroidal hyperTDs represent an early harbinger of GA, and, historically, GA is detected on color fundus imaging.^16^ However, these large persistent hyperTDs are identified by using en face optical coherence tomography (OCT) imaging.^3^^,^^17^^–^^19^ Large hyperTDs are defined as bright areas with a greatest linear dimension (GLD) of at least 250 µm on en face slabs beneath the RPE (sub-RPE slabs), segmented at 64 to 400 µm under the Bruch's membrane (BM). They correspond to increased penetration of light into the choroid due to attenuation or loss of the outer retina and RPE on B-scan images.^16^^,^^18^^,^^19^ Our previous publications have shown that, when these hyperTDs achieve a GLD of 250 µm, they have a persistence rate of 99.6%.^17^^,^^20^ Implementing the same en face sub-RPE imaging strategy, we have identified several high-risk OCT biomarkers that precede the formation of hyperTDs such as increased drusen volume measurements, increased area measurements of hyperreflective foci (HRF), and the presence of calcified drusen (CaD).^19^^,^^21^^–^^23^

Using the same swept-source OCT angiography (SS-OCTA) scanning strategy that we use to identify the high-risk OCT structural biomarkers, we are able to visualize and quantify the underlying CC.^24^^–^^33^ The flow signal is generated by using the optical microangiography algorithm.^34^ Previously, we used this imaging strategy to show age-related changes in the CC of normal eyes, and we correlated the growth rate of GA with the increase in CC flow deficits around the margins of GA as well as throughout the entire scan area.^28^^,^^35^^,^^36^

With an increasing focus on treating GA as early as possible to preserve as much vision as possible and with the appreciation that the current complement inhibitors only slow disease progression,^37^^,^^38^ we wanted to better understand whether the loss of the CC could be used as an angiographic biomarker to predict the onset of hyperTD so that therapy could be started before atrophy formed. To investigate the CC at the site of hyperTD formation, we used a novel grid strategy that we developed to track focal macular regions over time and correlate these structural changes with changes in CC perfusion.^33^ More recently, we published our updated strategy to quantify the CC, addressing the best compensation strategy to adjust for imaging artifacts that arise in AMD eyes and refining our ability to accurately measure flow deficits.^39^ In this retrospective longitudinal study, we used SS-OCTA scans to image intermediate AMD (iAMD) eyes and identify the onset of large hyperTDs and then quantify CCFDs prior to and after the onset of hyperTDs with the goal of determining the temporal relationship between CCFDs and hyperTDs.

## Materials and Methods

Patients diagnosed with AMD were enrolled in an ongoing prospective, observational SS-OCTA imaging study at the Bascom Palmer Eye Institute. This study was approved by the University of Miami Miller School of Medicine Institutional Review Board, and all participants signed an informed consent. The study was conducted in alignment with the tenets of the Declaration of Helsinki and the Health Insurance Portability and Accountability Act of 1996.

### Participants

A retrospective review of AMD subjects prospectively enrolled from April 2016 to June 2023 was conducted to identify eligible eyes diagnosed with iAMD, and this review resulted in a dataset of 171 eyes.^21^ The diagnosis of iAMD required the presence of at least one druse with a minimum diameter of 125 µm within a 5-mm fovea-centered circle. Follow-up time was required to be a minimum of 1 year, and the imaging frequency varied depending on the clinical progression and patient availability. Eyes with pre-existing exudative macular neovascularization, GA, or large hyperTDs were excluded, as well as those with diabetic retinopathy or other retinal diseases associated with drusen-like deposits such as Stargardt disease or vitelliform dystrophy. Eyes with significant vitreoretinal interface disease that distorted macular anatomy or with previous vitreoretinal surgery were also excluded.

### Imaging Protocol

All patients underwent fovea-centered 6 × 6-mm SS-OCTA imaging (PLEX Elite 9000; Carl Zeiss Meditec, Dublin, CA, USA) acquired by one of two trained imaging technicians. The SS-OCTA instrument has a central wavelength of 1050 nm and a speed of 100,000 A-scans per second, producing 500 A-scans per B-scan with a uniform 12-µm spacing between both A-scans and B-scans. Each B-scan is repeated twice at the same location to obtain the angiographic signal information and processed using the complex optical microangiographic algorithm of the instrument. Scans were reviewed for image quality and signal strength and excluded if the signal strength was below 7 or if significant motion artifacts were present. When several scans were available for an eye on the same date, the highest quality scan was selected.

### Visit Selection

**Figure 1.**
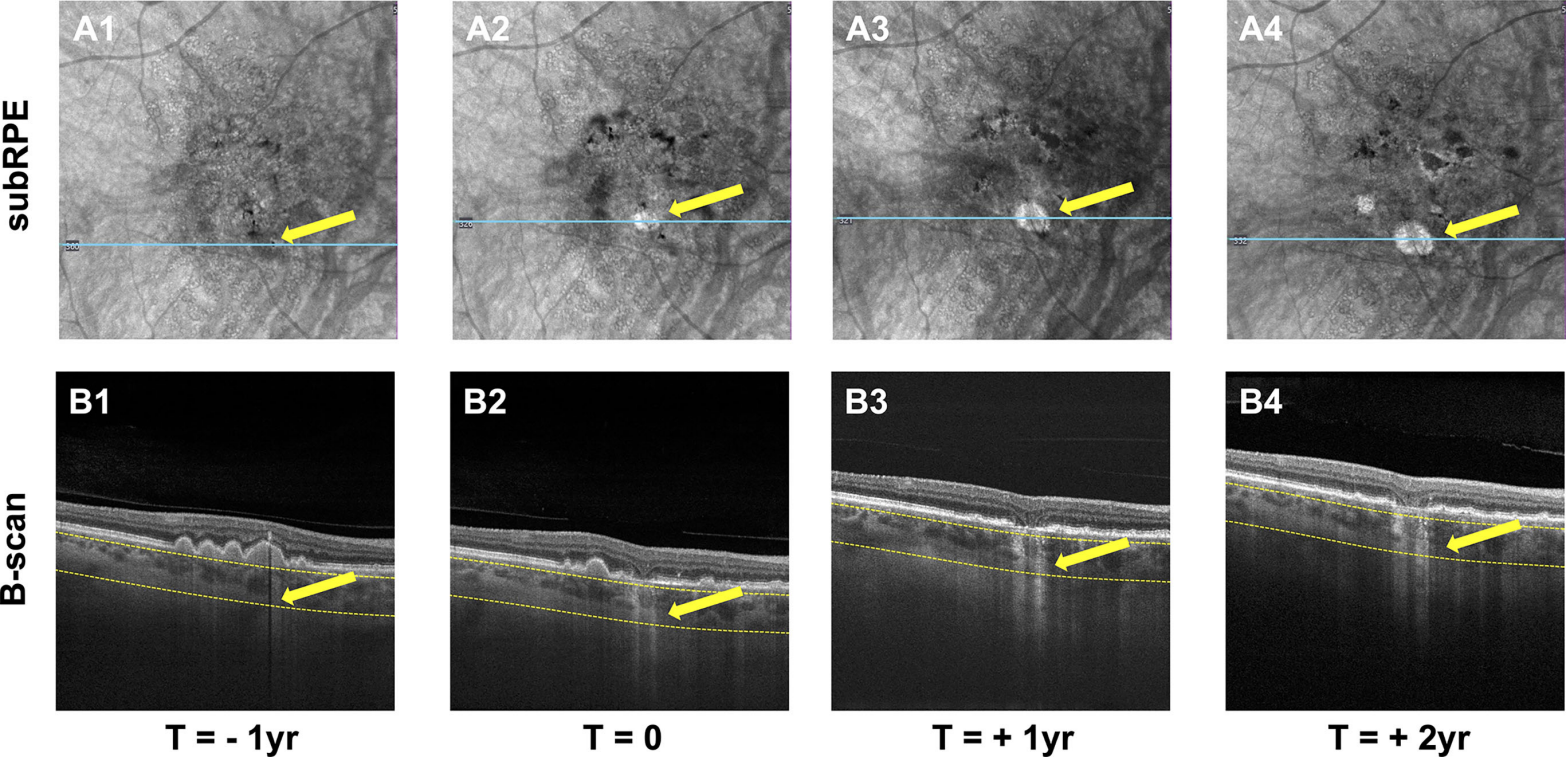
Selection of longitudinal visits before and after the onset of large choroidal hyperTDs. (**A1**–**A4**) En face structural SS-OCTA images generated using the sub-RPE slab positioned from 64 to 400 µm under the BM. The *arrows* identify the target area throughout the visits, whereas the *blue line* identifies the position of the B-scans shown in **B1** to **B4**. In **A2**, the arrow identifies the onset of the target large hyperTD defined as a focal bright area with a GLD of at least 250 µm and confirmed on the B-scan view in **B2** by the presence of choroidal hyperTD (*arrow*), with overlying outer retinal and RPE attenuation. The *dashed yellow lines* in the B-scans (**B1**–**B4**) identify the segmentation boundaries that are used to generate the en face sub-RPE slab. **A1** shows the visit at 1 year before the onset of the large hyperTD with the *arrow* identifying where the hyperTD will develop (**A2**). This is referred to as the target region. The corresponding B-scan view in **B1** confirms the absence of a large hyperTD in the target area indicated by the *arrow*. **A3** and **A4** show two more visits selected at 1-year and 2-year intervals after the onset of the large hyperTD, and the *arrow* indicates the presence of a large hyperTD in the target area corresponding to the choroidal hyperTDs, as shown in B-scans **B3** and **B4**.

From our database of 171 eyes with iAMD, we identified 82 eyes that developed large hyperTDs as defined by the presence of bright lesions with GLD of at least 250 µm on the sub-RPE en face slab image generated by using segmentation boundaries of 64 to 400 µm beneath the BM.^17^^,^^18^^,^^20^ The areas of en face focal brightness diagnosed as hyperTDs corresponded to regions with increased light penetration into the choroid due to attenuation or loss of the RPE, as confirmed on corresponding B-scans. The visit when a hyperTD was first identified was referred to as the date of hyperTD onset and designated as T = 0 (Figs. 1A2, 1B2). Additional visits were selected at 1-year intervals before hyperTD onset and after hyperTD onset (Figs. 1A1, 1A3, 1A4, 1B1, 1B3, 1B4). Only eyes with large hyperTDs that had available visits separated by 12 ± 3-month intervals were included.

### Exclusion Masks

**Figure 2.**
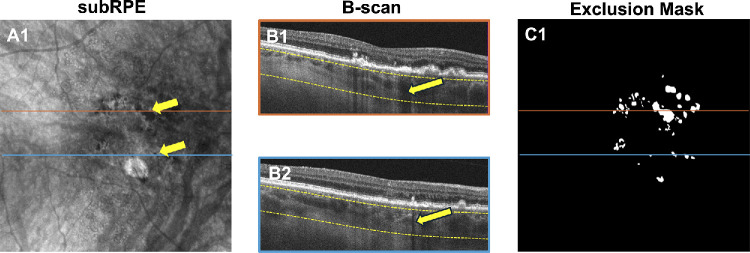
Creation of the exclusion mask used for the measurement of CCFDs. This figure depicts the same visit as Figure 1 in **A3** and **B3**. **A1** shows the structural en face sub-RPE slab image derived from SS-OCTA scans. The en face sub-RPE slab is defined by segmentation borders of 64 to 400 µm under the BM. *Yellow arrows* indicate choroidal hypoTDs appearing as dark foci on the en face sub-RPE image, whereas *color-coded lines* identify the position of the B-scans in **B1** and **B2**. B-scans from the color-coded **B1** and **B2** confirm the presence of a calcified druse (**B1**) and an intraretinal hyperreflective focus (**B2**), both casting a shadow identified by the *yellow arrows* which correspond to hypoTDs on the en face image (**A1**). The *yellow dashed lines* in **B1** and **B2** identify the segmentation boundaries of the sub-RPE slab. **C1** shows the total exclusion mask constructed by identifying all the hypoTDs from the en face sub-RPE image that have been confirmed by using the corresponding B-scans. All *white areas* represent hypoTDs that were excluded from the quantification of CCFDs.

Choroidal hypotransmission defects (hypoTDs) associated with HRF and CaD as confirmed on B-scans appeared as dark foci on en face sub-RPE images and corresponded to regions of decreased light penetration into the choroid that prevented visualization of the CC (Figs. 2A1, 2B1, 2B2).^21^^–^^23^^,^^40^ These areas cannot produce reliable CC quantification and must be excluded from the analysis. This was accomplished by the creation of manually outlined exclusion masks (Fig. 2C1).^41^^,^^42^ Exclusion masks were created for each visit and then combined at the end of the follow-up period so that the same regions were excluded throughout the study period. Areas of peripapillary atrophy were also included in these masks. If the hyperTD area was initially occupied by hypoTDs at the pre-onset visit, then that case was excluded due to the inability to adequately measure the CCFD percentage (CCFD%) values in these regions.

### Compensation Strategy

Drusen and other abnormalities of the RPE/BM complex that are prevalent in AMD eyes can produce focal areas of OCT signal attenuation in the CC layer, typically visualized as darker regions or shadowing on the en face CC structure slab. To minimize the effect of attenuation artifacts on CC flow quantification, we implemented a validated signal compensation strategy^25^^,^^26^^,^^28^^,^^30^^–^^32^ with additional updates recently published by Berni et al.^39^ This compensation strategy used a parameter gamma (γ) that is optimized for each scan to provide the highest homogeneity or lowest standard deviation of pixel illumination across the compensated CC structure slab.^39^ However, we found that in some cases high-frequency noise caused instability in the parameter optimization.^43^ In order to remedy this, a Gaussian filter (kernel size of 13 × 13 pixels and a sigma of 15) was applied before optimization.

### Masking HyperTDs Prior to Compensation

**Figure 3.**
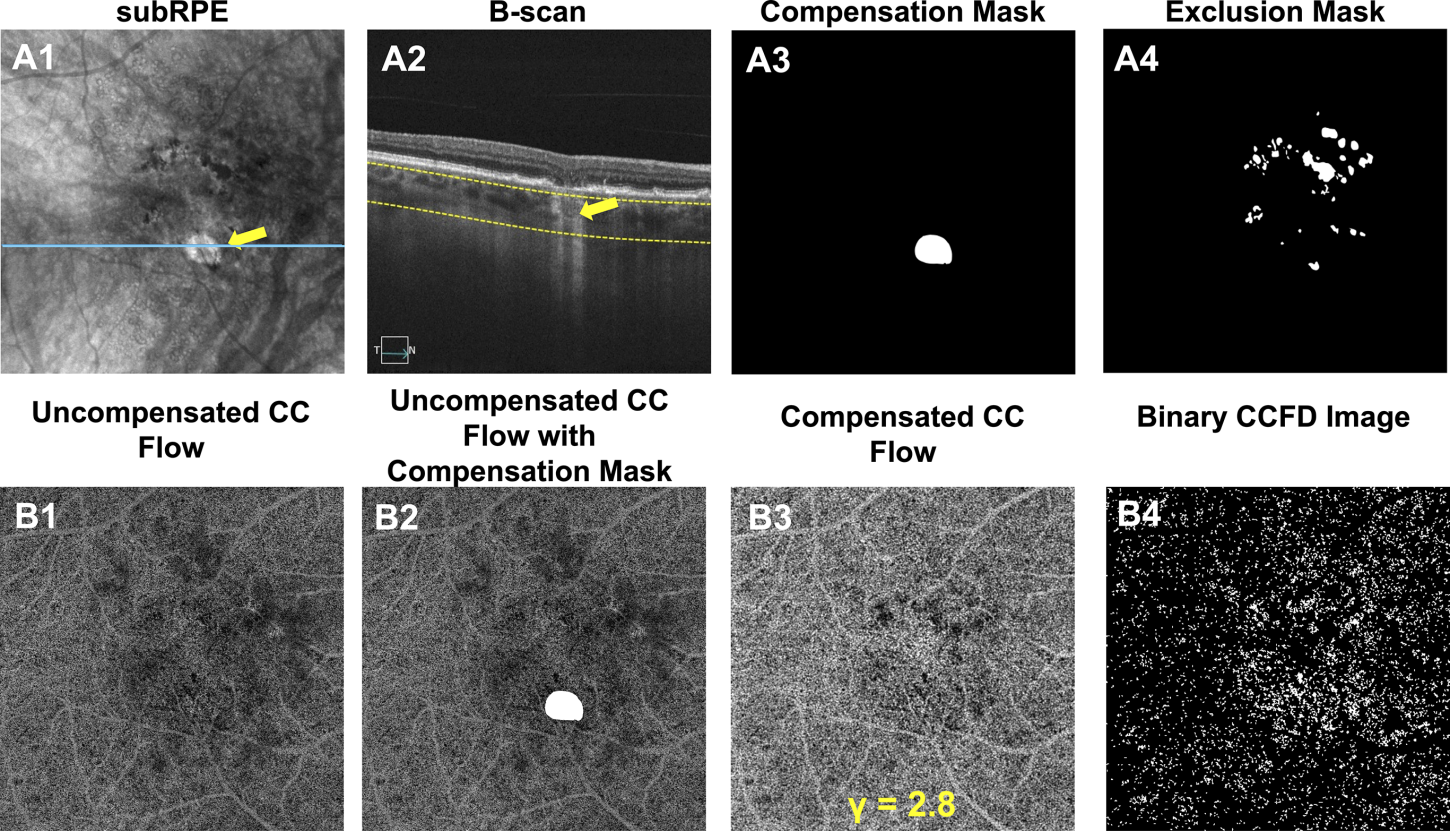
This figure illustrates CCFD quantification on the same visit as Figure 2. (**A1**) The en face sub-RPE slab with segmentation boundaries at 64 to 400 µm under the BM. The *yellow arrow* points to the target large hyperTD, defined as a focal region of increased brightness with a GLD of at least 250 µm. The *blue line* in **A1** indicates the position of the corresponding B-scan in **A2**, confirming the presence of a large choroidal hyperTD (*yellow arrow*) associated with attenuation of the outer retina and RPE. The *interrupted yellow lines* in **A2** depict the segmentation boundaries of the sub-RPE slab described above. (**A3**) Compensation mask generated by outlining all large hyperTDs identified on the respective visit. This mask is used to avoid compensation over areas of hyperTD, which would produce artifactually high CCFD% values as demonstrated by Berni et al.^39^ (**A4**) Exclusion mask generated in Figure 2, which was used to exclude these areas from the compensation and quantification analyses and applied prior to the production of the binary CCFD image. (**B1**) Uncompensated en face CC flow slab with segmentation boundaries of 4 to 20 µm under the BM. (**B2**) Areas of the uncompensated en face CC flow slab that will be compensated; the *white mask* outlines the hyperTD area that will not be compensated. (**B3**) Compensated en face CC flow slab after applying the optimal compensation value (γ = 2.8) while maintaining the hyperTD mask (**B2**), which was removed after compensation. (**B4**) Binary CCFD maps obtained using the Fuzzy C-Means global thresholding method on the compensated CC flow image. In this binary CCFD map, the *stippled white areas* represent flow deficits or CCFDs dotted over a *black background* of detectable flow.

HyperTDs correspond to areas with increased light penetration into the choroid due to the attenuation or loss of RPE over the CC. Berni et al.^39^ demonstrated that applying compensation indiscriminately to the bright areas of hyperTDs can lead to an excessive OCT signal reduction in the process of inversion and a corresponding artifactual increase of CCFD% values. Following these updated guidelines, we manually outlined all areas of hyperTDs and created a compensation mask that avoided compensation in these regions. After the compensation step was completed, these focal areas were unmasked and included in the CCFD% quantification step (Figs. 3A1–A3, 3B1–B3).

### CC Slab Signal Thresholding and Binarization

A semi-automated algorithm was used to select a 16-µm-thick slab positioned from 4 to 20 µm under the BM. The choice of this slab has been previously validated and corresponds to the anatomical location of the CC, taking into consideration the convolutional effect of the OCT instrument that artificially increases the CC thickness.^30^ The segmentation output of the algorithm was carefully reviewed for each B-scan and manually corrected within the software whenever necessary. Any cases where segmentation was significantly affected by the presence of nonexudative macular neovascularization or imaging artifacts were excluded.

The CCFD quantification algorithm applied an optimized compensation level previously described while excluding the areas of hyperTD, as outlined by the compensation mask (Figs. 3B1–B3). The exclusion mask corresponding to the hypoTDs was then applied (Fig. 3A4) and retinal vessel projection artifacts were automatically removed.^44^

The Fuzzy C-means global thresholding strategy was used to binarize the image as previously described, and all identified perfusion deficits with a size <24 µm (a threshold representing the physiological intercapillary distance) were removed.^24^^–^^26^^,^^30^^,^^33^^,^^35^^,^^45^ The resulting CCFD binary maps displayed flow deficits as white areas over a black background of detectable flow (Fig. 3B4).

### Box Characteristics and Exclusion Criteria

The target box dimensions were 74 × 74 pixels (approximately 0.9 × 0.9 mm), covering an area of 0.81 mm^2^. The box was always centered at the target using automated software. To ensure a reliable comparison of CCFD measurements across all grid boxes, we applied an empirical threshold to exclude any box in which more than 25% of the area was unavailable for quantification due to either pixel masking by the integrated exclusion mask or low pixel signal intensity. Based on our testing, we found an intensity level of 5 dB above the median background signal to be an appropriate threshold across all the cases, adapting similar approaches from the literature.^46^^,^^47^ To be included, a target box was required to meet this 25% threshold. However, if individual background boxes in the grid failed to meet this threshold, then they were excluded from the analysis. Additionally, all other boxes that contained non-target hyperTDs, significant imaging artifacts, or areas cropped due to the registration process were also excluded from the analysis.

### Grid Registration

**Figure 4.**
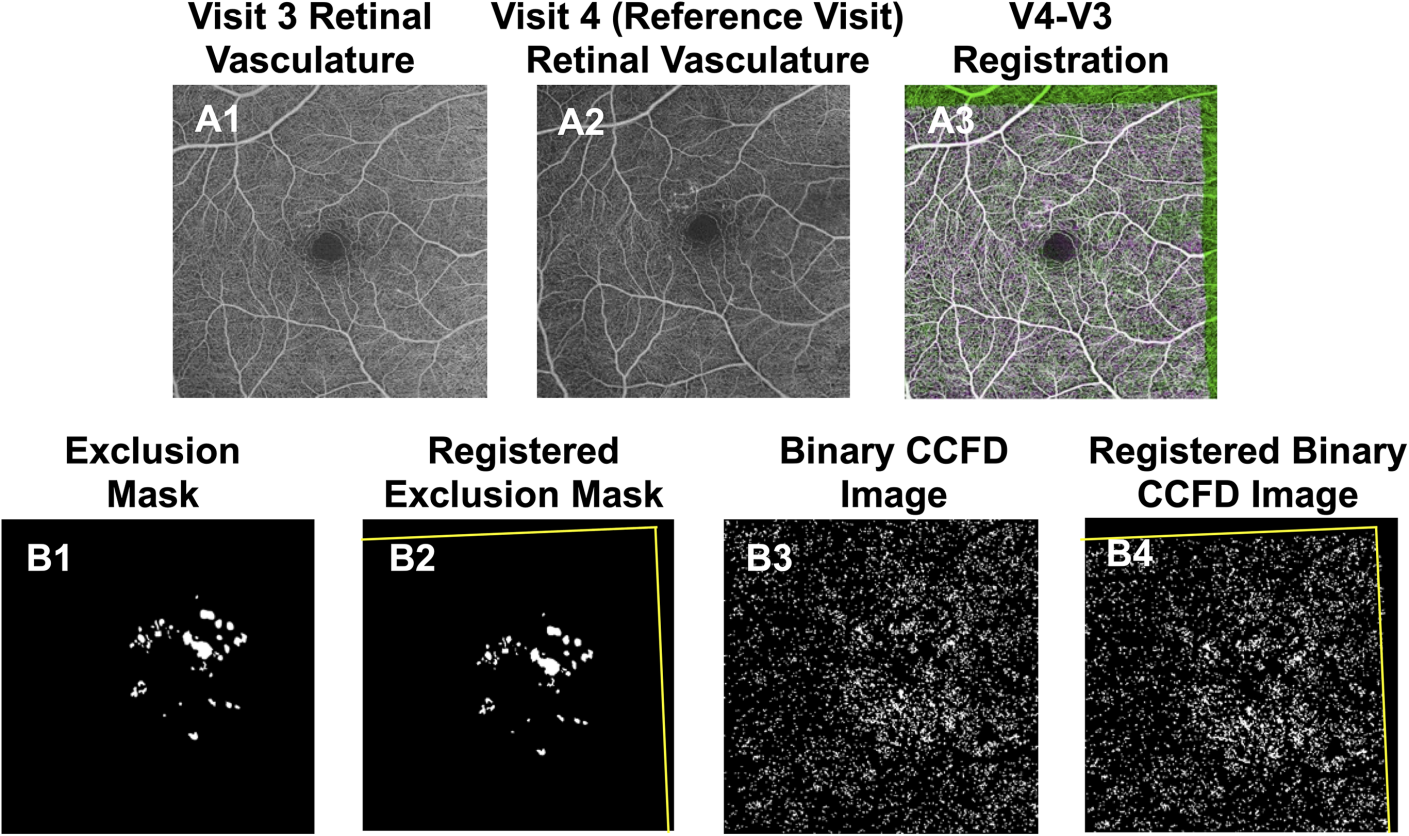
This figure illustrates the grid registration process for the measurement of CCFDs on the same visit as Figures 2 to 3. (**A1**, **A2**) The retinal vasculature images obtained using SS-OCTA. **A1** shows the retinal vasculature for the visit from Figures 2 and 3, corresponding to the third visit in the longitudinal follow-up, 1 year after the onset of hyperTD. **A2** shows the retinal vasculature from the fourth visit, 2 years after the onset of the large hyperTD. This final visit (**A2**) in the longitudinal four-visit series was designated as the registration reference. (**A3**) How the registration algorithm assessed the shift of vessel location between visit 3 and reference visit 4. Similarly, all of the other visits (visit 1 and visit 2) were registered to visit 4. (**B1**) The exclusion mask generated in Figure 2. (**B2**) The registered exclusion mask, generated by applying the recorded registration (**A3**) to the exclusion mask in **B1**. (**B3**) The binary CCFD image generated in Figure 3. (**B4**) The registered binary CCFD image, generated by applying the recorded registration (**A3**) to the binary CCFD image in **B3**.

**Figure 5.**
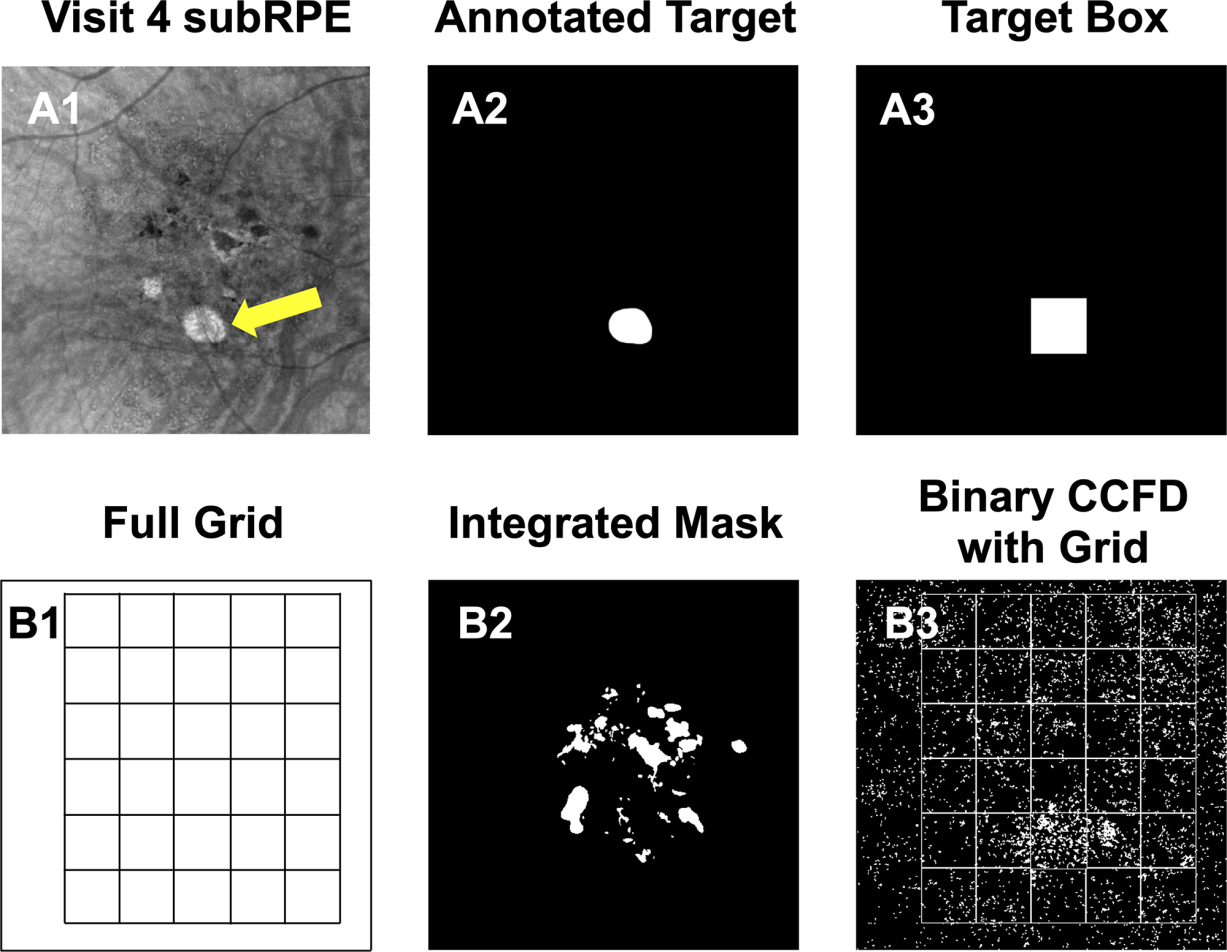
Grid registration step in the measurements of CCFDs. (**A1**–**A3**) The generation of the target box. **A1** shows the en face sub-RPE image from the reference visit (visit 4) with segmentation boundaries at 64 to 400 µm under the BM, and the *yellow arrow* indicates the target hyperTD. **A2** shows the annotation of the target hyperTD, and **A3** shows the 74 × 74-pixel target box (1 pixel = 12 µm) automatically generated by the registration software and centered around the annotated hyperTD. (**B1**) A grid of equally sized 74 × 74-pixel boxes created by the algorithm to span the imaged region. (**B2**) The final integrated mask, which is produced by merging the four individual registered exclusion masks. (**B3**) The final binary CCFD image obtained after application of the integrated mask from **B2** and overlaying the grid from **B1**. Selection of the final visit of the series (**A1**) as the registration reference allowed all target hyperTDs from prior visits to be aligned to the target of the final visit as annotated in **A1** to **A3**. In addition, the placement of the target and grid boxes from **A3** and **B1** on registered images ensured that the same areas were tracked by the same boxes throughout the follow-up, whereas application of the integrated mask (**B2**) ensured that the excluded regions were kept consistent across all binary CCFD images.

To reliably track and compare the changes in CCFDs in the same areas across visits, we used the same grid registration technique described by Hiya et al.^33^ The final visit from the four longitudinal visits was selected as the registration reference (Fig. 4A2). Registration was recorded by comparing the vessel locations from the en face retinal vasculature images of each visit to the reference visit (visit 4) (Figs. 4A1–A3), and this registration was applied to the respective binary CCFD images and exclusion masks (Figs. 4B1–B4). All individually registered exclusion masks were merged to form a final “integrated” mask, which was applied onto the registered binary CCFD images so that the same regions were excluded in each of the four visits (Fig. 5B2).

The target hyperTD from the final visit was manually annotated on the en face sub-RPE image, and the registration algorithm then centered a 74 × 74-pixel target box around this area (Figs. 5A1–A3). A grid of equally sized boxes was then automatically created, extending across the image until a row or column of complete boxes could no longer be accommodated (Fig. 5B1). This grid was overlaid onto the registered CCFD binary images, ensuring that the CCFD values in the target and background boxes were consistently tracked throughout the longitudinal follow-up (Fig. 5B3).

### Statistical Methods

Hiya et al.^33^ performed an analysis of this grid strategy and established that, for cases with hyperTDs, there is a 4.67% minimal detectable change (MDC) threshold for a single grid box.^33^ We therefore implemented a 5% threshold to detect a true change in CCFD% outside the test–retest variability limits when comparing the same target box across our four visits.

We defined a background box as any grid box that did not contain a target or non-target hyperTD in any of the four visits. If a target hyperTD extended beyond a single target box, then these boxes were considered as non-target hyperTD boxes and were excluded from the background box analysis. To further define this category, we classified background boxes into “adjacent” background boxes, defined as those immediately adjacent to any box containing a hyperTD in any of the four visits, and “non-adjacent” background boxes, defined by their location as not being immediately adjacent to any hyperTD box. The designated status of adjacent or non-adjacent was maintained unchanged across all four visits for any given box. Therefore, if a box was adjacent to a hyperTD by visits 3 and 4, but not at visits 1 or 2, it was categorized as an adjacent box throughout our analysis.

Group differences among all boxes in each of the above categories (target, background, adjacent, non-adjacent) were assessed across the four visits by estimating the mean differences using linear mixed models with random intercepts for patients, eyes clustered within patients, and grid boxes clustered within eyes. Tukey's method was used to adjust for multiple comparisons. A random intercept was removed from a model only if the associated variance component was estimated at zero, resulting in a singular fit. All statistical analyses were performed using R 4.5.0 (R Foundation for Statistical Computing, Vienna, Austria) with the emmeans, lme4, and tidyverse packages. *P* < 0.05 was considered statistically significant for this study.^48^^–^^51^

## Results

We identified a total of 34 eyes from 29 patients for this study after excluding all cases that did not have visits available in a 12 ± 3-month interval, after excluding eyes in which the areas of the hyperTDs were entirely occupied by hypoTDs, and after excluding eyes in which the CC segmentations were significantly affected by the presence of macular neovascularization or other artifacts. Additionally, seven eyes were excluded from further analysis because the target boxes did not meet the 25% threshold discussed above, resulting in a final sample of 27 eyes from 24 patients. Each eye was imaged at four visits separated by standard intervals of 12 ± 3 months with an average follow-up duration of 36.0 ± 2.1 months (median, 37 months; range, 32–39). The mean age of the 24 patients at baseline was 74.0 ± 7.9 years (range, 53–90), and 16 of them were female (66.7%). All eyes had axial lengths ≤ 26 mm. Figure 1 shows a representative example of four visits of the longitudinal follow-up of changes in CCFD% measurements. The baseline status for all eyes at the first visit was intermediate dry AMD with no history of treatment, and this was designated as the pre-hyperTD onset visit (Fig. 1A1). At the second visit, all eyes developed at least one hyperTD, and this visit was designated as the hyperTD-onset visit (Fig. 1A2). Between the 1-year pre-onset visit and the hyperTD-onset visit, most of the patients had more follow-up visits that were not included in this study. After reviewing these visits, we found that the duration between the last visit where a hyperTD was not documented prior to its onset was on average 5.04 ± 3.36 months (range, 1–11). The final visit was at 2 years post-onset of the hyperTD. By this final point in the follow-up, 26 eyes remained as dry AMD, two of them had started intravitreal pegcetacoplan injections, and one eye had converted to wet AMD and was initiated on anti-vascular endothelial growth factor (VEGF) injections. Among the 27 eyes, four eyes changed status from phakic to pseudophakic during the monitoring interval.

### Grid Box Exclusion Results

A total of 23 background boxes in the 27 study eyes did not qualify according to the 25% threshold criteria discussed above and were excluded from the analysis. Additionally, 24 individual background boxes were excluded due to artifacts or cropping associated with the registration shift, and 95 were excluded due to the presence of non-target hyperTDs. In summary, 27 target boxes and 587 background boxes from 27 eyes were included in this analysis.

### Large HyperTD Characteristics

The average target hyperTD area at the onset of the hyperTD across 27 eyes was 0.10 ± 0.07 mm^2^ (median = 0.09 mm^2^; range, 0.04–0.35). At the visit 1 year after the onset of hyperTD, the average target hyperTD area increased to 0.27 ± 0.44 mm^2^ (median = 0.12 mm^2^; range, 0.04–2.20), and at the visit 2 years after hyperTD onset, the average target hyperTD area was 0.40 ± 0.58 mm^2^ (median = 0.16 mm^2^; range, 0.04–2.56).

### Change in CCFD% Measurements Across Four Visits

**Figure 6.**
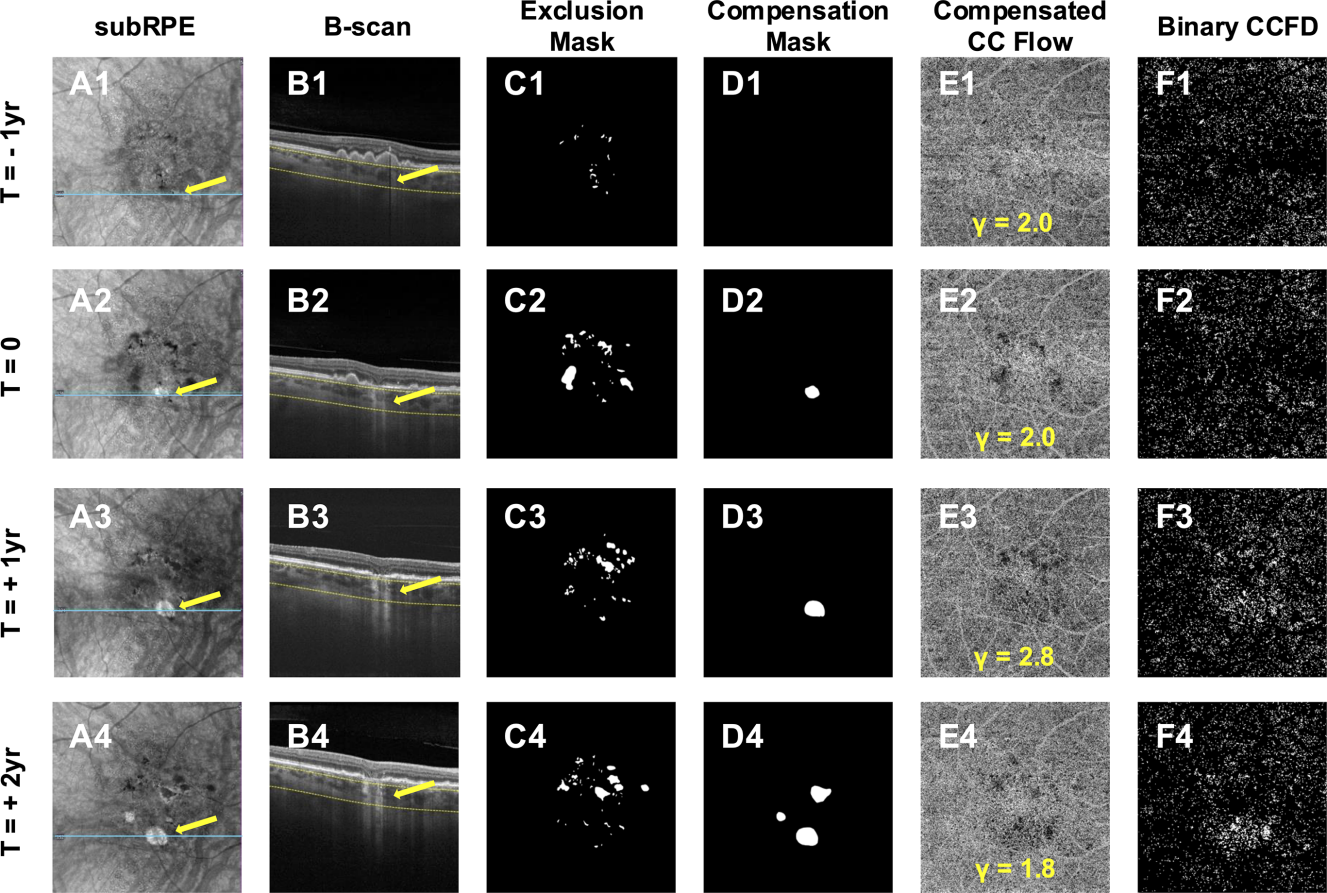
Measurement of CCFDs at sequential visits. This figure illustrates the complete workflow on all the visits of an AMD eye that developed a large choroidal hyperTD from Figures 1 to 5. (**A1**–**A4**) The en face sub-RPE slab with segmentation boundaries at 64 to 400 µm under the BM imaged using SS-OCTA in a 6 × 6-mm pattern. The target area is indicated by the *arrows*; in **A1**, it indicates the absence of hyperTD at 1 year prior to its onset, whereas in **A2** to **A4** they indicate the presence of a hyperTD, defined as a focal area of increased brightness with a GLD of at least 250 µm. The *blue line* on the en face sub-RPE images is placed over the target area and corresponds to the location of the B-scans in **B1** to **B4**. (**B1**–**B4**) The *yellow interrupted lines* show the segmentation boundaries of the sub-RPE slab, and the *yellow arrows* correspond to the target areas in **A1** to **A4**, indicating the absence of a hyperTD in the target region (**B1**) and then the presence of hyperTD in **B2** to **B4**. **(C1–C4**, **D1–D4**) Exclusion masks and compensation masks respectively for each visit. Areas covered by the exclusion mask were not included in the quantification of the CCFDs, whereas areas covered by the compensation masks were not compensated but were quantified. (**E1**–**E4**) The en face compensated CC flow images with segmentation boundaries of 4 to 20 µm under the BM, and the optimal γ parameter implemented for each visit is shown at the bottom of the scan. (**F1**–**F4**) The binary CCFD images obtained after applying the Fuzzy C-Means global thresholding on the compensated CC flow images, with *stippled white areas* indicating absence of flow in the CC and the *black background* representing detection of flow.

**Figure 7.**
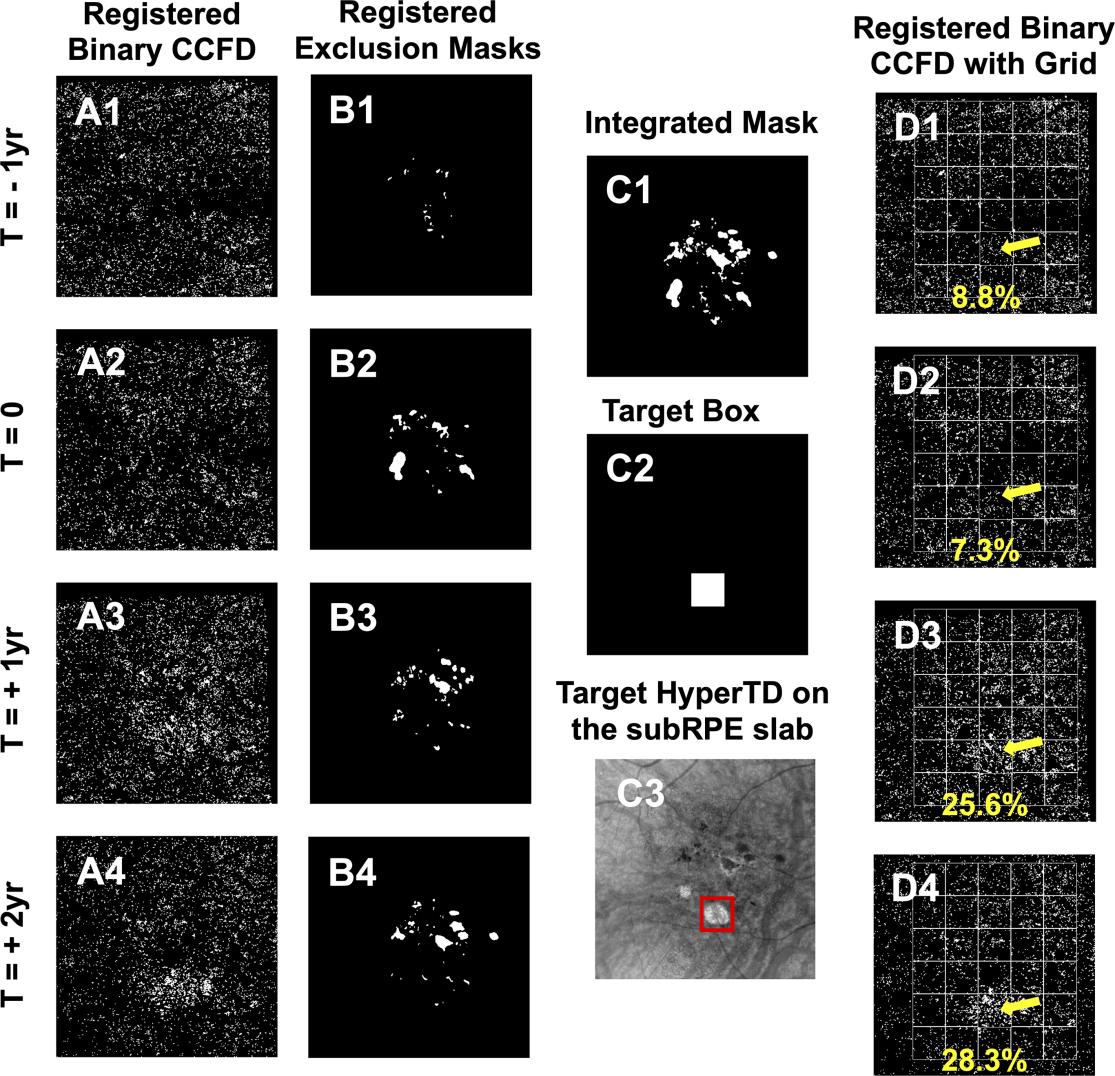
Overall grid workflow strategy for the longitudinal measurements of CCFDs. This figure illustrates the complete workflow on all of the visits of an AMD eye that developed a large choroidal hyperTD from Figures 1 to 6. (**A1**–**A4**, **B1**–**B4**) The registered binary CCFD images and registered exclusion masks obtained by aligning each visit to the final visit, the designated registration reference, as described in Figure 4. (**C1**) The integrated mask, generated by merging all the individual registered exclusion masks, as described in Figure 5. The integrated mask was applied on each registered binary CCFD image to ensure that the same regions were excluded from CCFD quantification across all visits. (**C2**) The target box. (**C3**) The location of this target box centered on the target hyperTD at the registration reference visit as described in Figure 5. (**D1**–**D4**) The registered binary CCFD images after application of the integrated mask with the grid overlay. Registration of each visit to the final reference visit ensured that all boxes would consistently track the CCFD values of the same region throughout the longitudinal follow-up. The *yellow arrow* indicates the target box, and the values at the bottom of each image show the target CCFD% values. No significant difference above the previously established 5% MDC of normal test–retest variability was observed between the visit at 1 year prior to hyperTD onset (**D1**) and at the onset of hyperTD (**D2**), but a marked rise was observed at 1 year (**D3**) and 2 years (**D4**) after the onset of hyperTD, with values exceeding the 5% change consistent with a real change compared with the first visit in **D1**.

Figures 6 and 7 illustrate an example of the complete workflow for the longitudinal follow-up of CCFD% measurements starting with the selection of four visits, from 1 year pre-onset to 2 years post-onset of the hyperTD, generation of masks, compensation, binarization, registration, and application of the grid strategy. Figures 7D1 to 7D4 show the target box CCFD% values for the case depicted in Figures 1 to 6. No significant difference in CCFD% values was found between the visit 1 year prior to the hyperTD onset (8.8%) and the onset of hyperTD (7.3%). A marked increase exceeding the 5% MDC was observed at 1 year (25.6%) and at 2 years (28.3%) after the onset of the hyperTD. Qualitatively, this change can be appreciated by an increase in the stippled white areas within the target box region in Figures 7D3 and 7D4 (yellow arrow).

**Figure 8.**
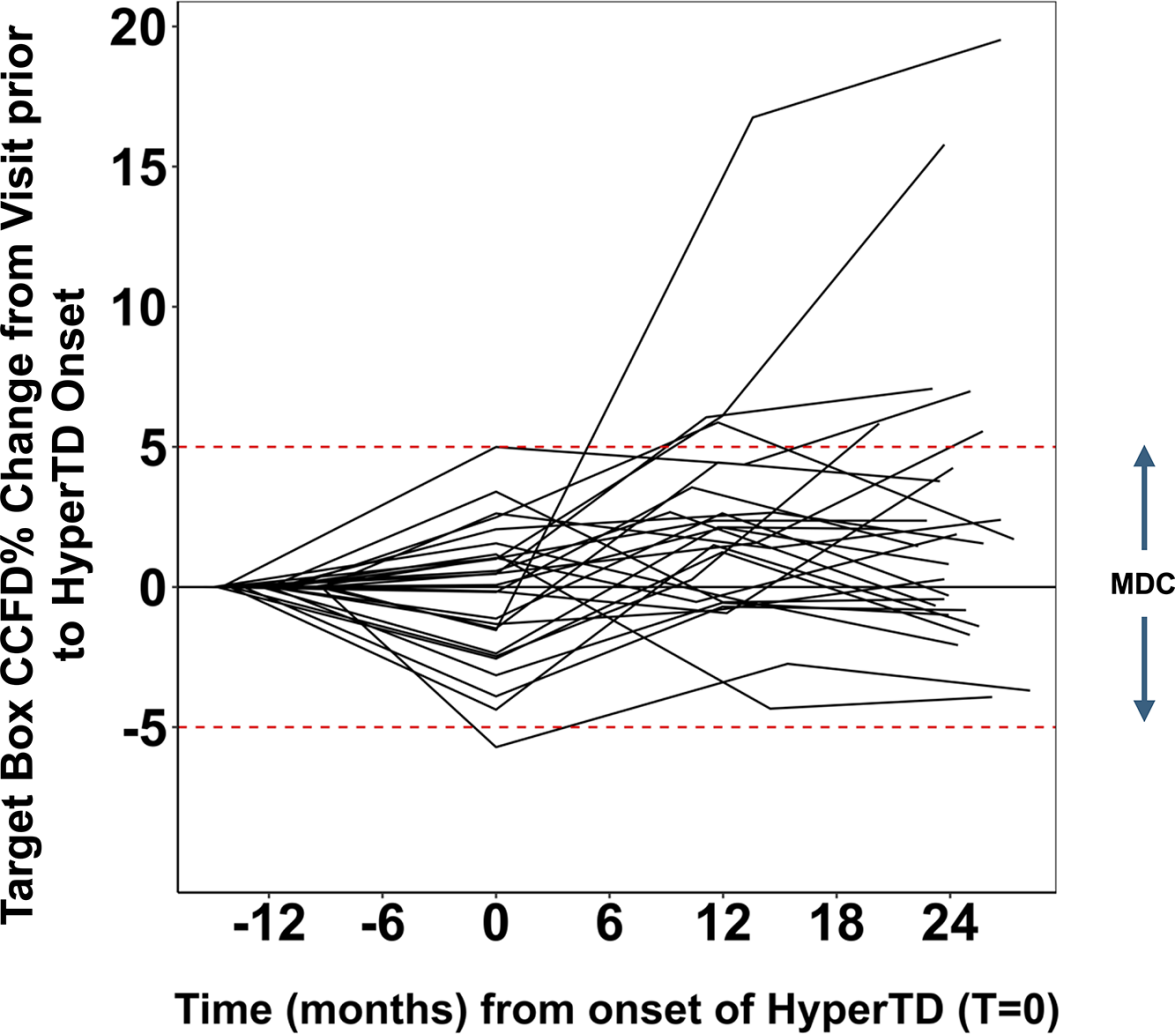
CCFD measurements over four visits for 27 targeted large choroidal hyperTDs from 27 AMD eyes. The graph shows the change in CCFD% values at the target box throughout the monitoring time. The *y*-axis represents the change in CCFD% in the target box between each follow-up visit and the visit prior to hyperTD onset; the *x*-axis represents the time in months from the onset of hyperTDs marked by T = 0; and the *red dashed lines* mark the 5% MDC range. No targets showed a marked rise above 5% between the onset of hyperTDs and the visit 1 year prior to the onset. At 2 years after the onset of hyperTDs, six targets exceeded the 5% MDC, with only two of them showing marked increases (>15%).

Figure 8 shows the changes in the target box CCFD% values across the four visits for all 27 eyes. There were no marked changes beyond the 5% MDC between the pre-hyperTD visits 1 year before the hyperTD onset and at the time of the hyperTD onset. However, by the end of the monitoring interval, at the visit 2 years post-onset of the hyperTD, six targets exceeded the 5% MDC, with two of them showing marked increases of 19.5% and 15.8%. These two cases proved to be the exception, as most of the cases did not exceed the 5% cutoff for a change outside the test–retest variability.

### Distribution of CCFD% Values Across Four Visits

**Figure 9.**
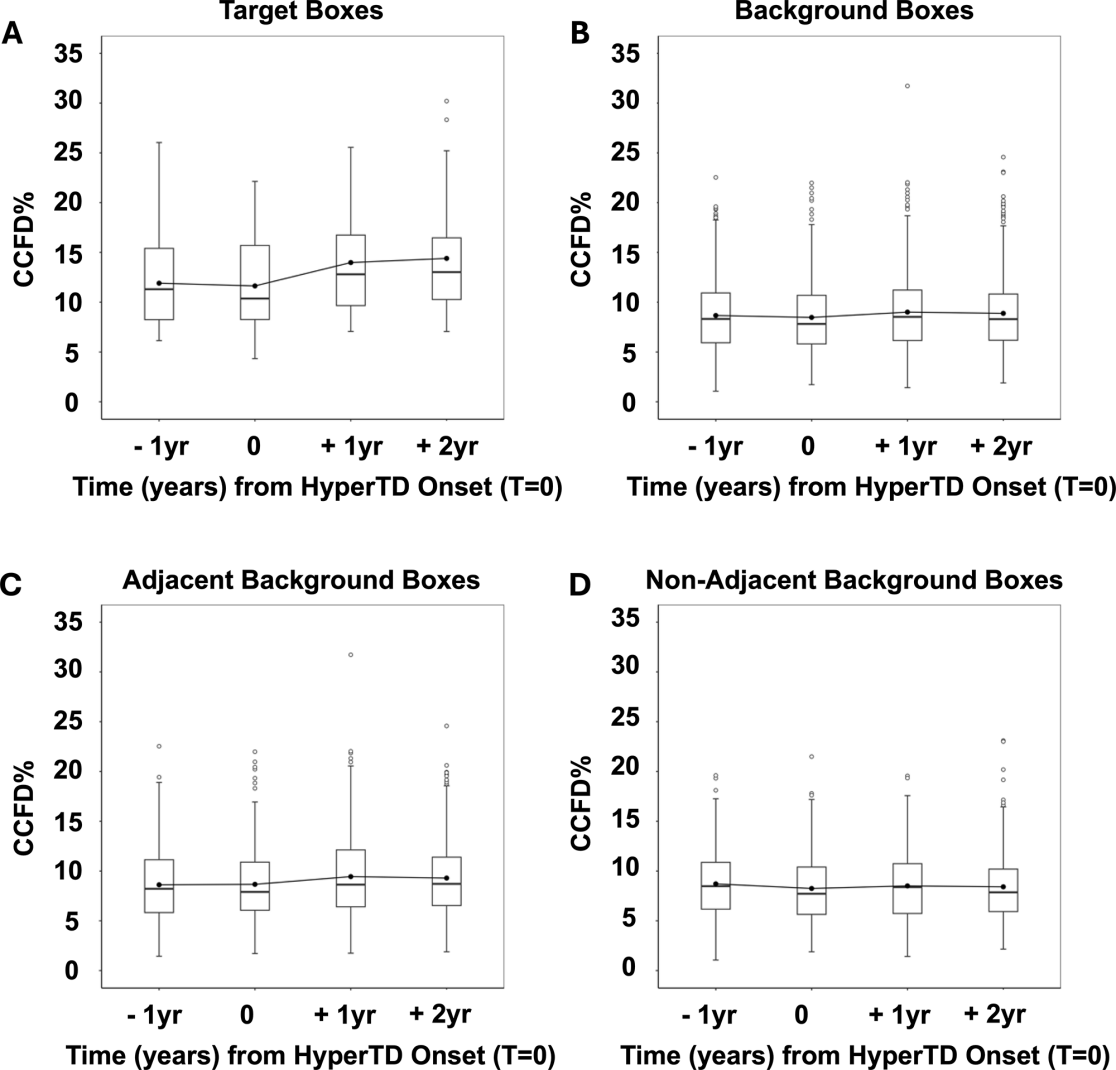
CCFD measurements in grouped target boxes, background boxes, adjacent background boxes, and non-adjacent background boxes across all 27 eyes using box-and-whiskers plots. The *y*-axis represents the CCFD% values, and the *x*-axis represents the nominal time in years for each visit from hyperTD onset (marked by T = 0). Each *box* represents the interquartile range (IQR). The *solid circle* within each box represents the mean, the *horizontal line* represents the median, and the whiskers extend to values within ±1.5 × IQR. Outlier points located beyond the whiskers are represented by *circles*. (**A**) The CCFD% distributions across 27 target boxes at each time point relative to the large hyperTD onset. (**B**) The CCFD% distributions across all background boxes at each time point relative to the large hyperTD onset. Background boxes are defined by the absence of large hyperTDs across all the visits. (**C**) The CCFD% distribution in adjacent background boxes, defined by their location immediately adjacent to any box containing a large hyperTD (target and non-target hyperTDs) in any of the visits. (**D**) The CCFD% distribution of non-adjacent background boxes, defined by their location not immediately adjacent to any large hyperTD box in any of the visits.

Figure 9A shows the distribution of target box CCFD% values per visit across all eyes. In the 27 target boxes, there was no statistically significant difference in the mean CCFD% values between the visits 1 year pre-onset of the hyperTDs compared with the visit at the onset of hyperTDs (estimated mean difference [EMD] = −0.27%; 95% confidence interval [CI], −1.72 to 1.18; *P* = 0.99) (Table 1). However, there were significant differences in mean CCFD% values when comparing the visits at 1 year and 2 years post-onset of hyperTD to the hyperTD onset visit (1 year: EMD = 2.35%; 95% CI, 0.89–3.80; *P* < 0.001; 2 years: EMD = 2.76%; 95% CI, 1.31–4.22; *P* < 0.001) (Table 1). It should be noted that the higher end of the whiskers range in the visit pre-onset of hyperTD reached a maximum of 26.0%, representing the only target box with a CCFD% that exceeded a CCFD% of 20% at the initial visit. This case also represents the maximum value of the whiskers range in the second visit at the onset of the hyperTD with a CCFD% of 22.1%, again the only one exceeding 20%, whereas by the third and fourth visits at 1 year and 2 years post-onset of hyperTDs CCFD% values in the 20s% were reached by five cases. Finally, the two outlier points with CCFD% values of 28.3% and 30.2% seen in the final visit at 2 years post-onset of hyperTDs correspond to the two cases with marked CCFD% change in Figure 8.

**Table 1.** Mean Differences in CCFD% Measurements Between Target Boxes at Different Time Points Compared With the Onset of Large Choroidal HyperTDs

	Boxes, *n*	EMD (95% CI)	*P*
1 y prior to hyperTD onset	27	−0.27% (−1.72 to 1.18)	0.99
1 y after hyperTD onset	27	2.35% (0.89 to 3.80)	<0.001
2 y after hyperTD onset	27	2.76% (1.31 to 4.22)	<0.001

Across all background boxes (Fig. 9B) there was no significant difference between the mean CCFD% values when comparing the pre-onset of hyperTD visit to the onset of hyperTD (EMD = −0.19%; 95% CI, −0.50 to 0.12; *P* = 0.44) (Table 2); however, there were significant differences when comparing the visits at 1 year and 2 years post-onset of hyperTDs to the onset of hyperTDs (1 year: EMD = 0.53%; 95% CI, 0.22−0.84; *P* < 0.001; 2 years: EMD = 0.41%; 95% CI, 0.10−0.72; *P* = 0.003) (Table 2). We observed a greater number of outliers in Figure 9B when compared to Figure 9A, which might be expected due to the markedly higher number of data points: specifically, 587 background boxes versus 27 target boxes.

**Table 2.** Mean Differences in CCFD% Measurements Between Background Boxes at Different Time Points Compared With the Onset of Large Choroidal HyperTDs

	Boxes, *n*	EMD (95% CI)	*P*
1 y prior to hyperTD onset	587	−0.19% (−0.50 to 0.12)	0.44
1 y after hyperTD onset	587	0.53% (0.22 to 0.84)	<0.001
2 y after hyperTD onset	587	0.41% (0.10 to 0.72)	0.003

The background boxes were divided into two separate classes according to their proximity to any hyperTD in the grid across all visits. Background boxes immediately adjacent to any hyperTD box at any single visit in the follow-up were referred to as “adjacent,” whereas the remaining background boxes were referred to as “non-adjacent,” resulting in a division of 311 adjacent and 276 non-adjacent boxes. Interestingly, adjacent boxes followed the same pattern as the target and background boxes, with the visit at 1 year pre-onset showing no significant difference from the visit at the time of hyperTD onset (EMD = 0.05%; 95% CI, −0.42 to 0.52; *P* = 1.0) (Fig. 9C; Table 3), whereas the visits at 1 year and 2 years post-onset of hyperTD showed a significant difference to the onset of hyperTDs (1 year: EMD 0.77%; 95% CI, 0.30–1.25; *P* <0.001; 2 years: EMD = 0.63%; 95% CI, 0.16–1.10; *P* = 0.003) (Table 3). However, the non-adjacent boxes present a different pattern in which the pre-onset and 1-year and 2-year post-onset visits did not display any significant differences compared with the hyperTD onset visit (pre-onset: EMD = −0.46%; 95% CI, −0.96 to 0.04; *P* = 0.08; 1 year: EMD = 0.26%; 95% CI, −0.24 to 0.76; *P* = 0.63; 2 years: EMD = 0.17%; 95% CI, −0.34 to 0.67; *P* = 0.92) (Fig. 9D; Table 4). This latter finding suggested that the changes in CCFD% values after the onset of hyperTDs depend on proximity to hyperTDs, with areas further away not affected to the same extent.

**Table 3.** Mean Differences in CCFD% Measurements Between Adjacent Background Boxes at Different Time Points Compared With the Onset of Large Choroidal HyperTDs

	Boxes, *n*	EMD (95% CI)	*P*
1 y prior to hyperTD onset	311	0.05% (−0.42 to 0.52)	1.0
1 y after hyperTD onset	311	0.77% (0.30 to 1.25)	<0.001
2 y after hyperTD onset	311	0.63% (0.16 to 1.10)	0.003

**Table 4.** Mean Differences in CCFD% Measurements Between Non-Adjacent Background Boxes at Different Time Points Compared With the Onset of Large Choroidal HyperTDs

	Boxes, *n*	EMD (95% CI)	*P*
1 y prior to hyperTD onset	276	−0.46% (−0.96 to 0.04)	0.08
1 y after hyperTD onset	276	0.26% (−0.24 to 0.76)	0.63
2 y after hyperTD onset	276	0.17% (−0.34 to 0.67)	0.92

Three of the 27 eyes received treatment after the onset of hyperTD; one eye received anti-VEGF therapy 12 months after the onset, and two eyes received pegcetacoplan at 3 months and 17 months after the onset, respectively. We re-calculated the post-treatment group statistics and found similar results for the target boxes, where there was no statistically significant difference prior to the hyperTD onset but a statistically significant increase in mean CCFD% at 1 year and 2 years after the onset. When assessing the total background and adjacent background boxes, there was no difference prior to the onset of hyperTD but but a significant increase was noted at 1 year post-onset. This significance was lost at 2 years after the onset; however, we could attribute this to the variability in the number of background boxes with a reduction in their number after exclusion by the final visit. Finally, the non-adjacent background boxes showed the same trend as before exclusion with no significant difference throughout visits.

## Discussion

An important topic of debate has focused on the respective roles of the RPE and the CC in initiating the cascade of events in eyes with iAMD that ultimately result in the formation of GA. In this study, we retrospectively investigated longitudinal SS-OCTA scans to understand the temporal and spatial associations between changes in CC perfusion, as determined by measuring CCFD%, and the appearance of choroidal hyperTDs. Our results show that longitudinal within-eye comparisons of CCFD% measurements reveal no marked increases in CCFDs that can be distinguished from test–retest variability before the onset of hyperTDs to the time of hyperTD onset. At 1 year and 2 years post-onset, we found that only a minority of eyes developed decreased CC perfusion at the site of the hyperTD target boxes. We also observed no significant differences in mean CCFD% compared with the pre-onset visit of hyperTDs when we grouped all of the target boxes positioned at the hyperTD site. However, the grouped data did show a mean increase at both annual post-onset visits compared with the visit at the time of hyperTD onset. A smaller increase in mean CCFD% after the hyperTD onset was also observed in the background boxes not affected by hyperTDs. When we subdivided the background boxes into those adjacent to hyperTDs and those non-adjacent, the increase in CCFD% post-onset remained statistically significant only in the hyperTD-adjacent boxes, suggesting that this latter category may be driving the mean increase in the overall background CCFD% and the change in CCFD% is more of a regional phenomenon and not just localized to the focal areas where the hyperTDs are located. Finally, we must note that the overall increasing trend in the grouped data could be due to a minority of cases, as the individual data show a large proportion of targets not reaching the 5% MDC throughout the follow-up.

Taken together, both individual and grouped analyses indicated that CCFDs remain stable before the onset of hyperTDs, but the target boxes and the boxes immediately adjacent to the hyperTD boxes showed an increase in CCFD% after the onset of hyperTDs. The totality of the results to date would suggest that significant changes in CC perfusion, as measured using the CCFD%, do not drive the appearance of hyperTDs, but rather, when the hyperTDs form, decreased CC perfusion develops at and near the sites of the hyperTDs.

This pattern of CC involvement supports the possibility of a two-stage pathogenic process. In this model, the initial stage may be driven by dysfunction or degeneration of the RPE/photoreceptor complex, while CC perfusion remains largely unaffected. In the subsequent stage, when RPE damage reaches a critical threshold and fails to produce adequate trophic support for the CC and/or there is decreased choroidal perfusion, CC perfusion decreases, further accelerating hyperTD expansion. This proposed sequence has implications for clinical trial designs. Specifically, CCFD% measurements may not serve as a meaningful biomarker for identifying cases at risk for the onset of hyperTDs. CCFD% measurements may be a better biomarker for interventions aimed at improving CC perfusion and limiting the growth of established hyperTDs in nonexudative AMD. This interpretation of our results is consistent with our previous report showing that increased CCFD% measurements around the margins of GA and throughout the entire scan area correlated with the growth rates of GA.^36^

Historically, histology has been widely used to investigate the RPE–CC mutualistic relationship and its subsequent degeneration. Several studies have described the loss of CC associated with normal aging in healthy eyes^52^^,^^53^^,^^54^ and in AMD eyes, where the degree of CC attenuation appeared to be associated with the severity of AMD.^52^^,^^55^ McLeod et al.^6^ reported a linear relationship between RPE and CC loss in GA suggesting that CC injury was secondary to RPE loss with residual CC remaining in areas of complete RPE loss, a hypothesis also promoted by Lutty et al.^5^ Grebe et al.^56^ and Edwards et al.^57^ further described the ultrastructure of the remaining CC in RPE atrophy as displaying extremely constricted vasculature with reduced fenestrations, suggesting that these changes were secondary to GA.

An opposing study by Biesemeier et al.^54^ showed that, in the “transition zone” at the margins of GA, the CC appeared more damaged than the RPE, leading them to conclude that this transition zone must be the site of first injury. In support of the vascular cause behind AMD progression, Mullins et al.^59^ demonstrated decreased CC density and increased ghost vessels associated with sub-RPE deposits, including drusen, and Lengyel et al.^58^ localized these deposits to areas of decreased vascularity also known as intercapillary pillars.

Although the above studies have provided compelling evidence for either sequence of events, it has been difficult to settle on a unifying mechanism strictly based on histopathology due to small sample sizes, lack of longitudinal data, potential tissue damage in the processing steps, and the possibility that the changes observed in eyes with AMD could be confused with CC changes that occur in normal aging.^35^^,^^59^^,^^60^ As Mullins et al.^59^ cautiously acknowledged, these experiments cannot detangle the cause from the effect. It could be that the RPE damage and the presence of deposits reduce the VEGF signaling necessary to maintain the CC. Alternatively, an initial vascular injury would impair the removal of debris, leading to deposit formation and ischemic injury to the RPE.

The advent of OCTA imaging of the CC provided a powerful new tool to investigate this question.^15^^,^^26^ Studies relying on commercially available spectral-domain OCTA (SD-OCTA) instruments support the histological findings of age-related CC changes^61^ and report markedly high CCFD% values in eyes with AMD, routinely exceeding 40%,^62^^–^^64^ as well as increased CCFD% values preceding the development of atrophy.^62^ Although investigations using SS-OCTA imaging have confirmed the presence of CC impairment associated with aging and AMD eyes,^29^^,^^31^^,^^35^^,^^65^^,^^66^ our current results do not replicate the reported high range of CCFD% measurements preceding disease progression in AMD, particularly the association with hyperTD formation seen in SD-OCTA investigations.

The key strengths of our quantification of the CC could explain why our results are different, such as the use of SS-OCTA imaging with a longer wavelength of 1050 nm as compared with 840 nm in SD-OCTA, resulting in decreased signal roll-off due to the better penetration of the SS-OCTA 1050-nm wavelength through the highly light-scattering RPE complex and better choroidal light penetration in the areas of RPE elevation such as drusen.^26^^,^^29^ Furthermore, the higher laser power provides better signal-to-noise ratio, and the faster scanning rate and denser scan pattern of 100,000 A-scans per second facilitated higher image quality.^67^^–^^69^ In addition, improved visualization of the CC was achieved by using the selection of an anatomically correct CC slab with improved strategies to address artifacts that lead to overestimating the loss of CC perfusion, such as in the presence of calcified drusen, hyperreflective foci, and inadequate compensation strategies, particularly under drusen.^30^^,^^39^^,^^42^

SS-OCTA imaging of CCFDs was achieved by applying the recommended segmentation boundaries for a slab that included the CC as described by Chu et al.^30^ This slab respected the physiological location of the CC, while avoiding overlap with the underlying choroidal vessels.^30^^,^^70^^,^^71^ All scans were reviewed and manually edited where necessary to prevent segmentation artifacts. In addition, diligent effort was made to avoid artifactual increases in the measurement of CCFDs by masking calcified drusen and HRF while avoiding compensation of hyperTDs.^39^^,^^42^ An optimized compensation strategy was implemented to ensure that signal attenuation was addressed to an appropriate level for the drusen volume of each individual scan while avoiding over-compensation.^30^^,^^39^ Moreover, thresholding and binarization was performed using the Fuzzy C-Means global thresholding as described by Chu et al.^24^^,^^36^

Using our novel grid registration strategy,^33^ we were able to track the same target and background boxes across visits and determine the changes in CCFD% measurements before and after the hyperTDs formed. Although different eyes had varying amounts of masking, we only included a grid box if at least 75% of the box area was unobstructed due to masking or poor signal quality. We first analyzed CCFD% changes in each target box longitudinally on an individual eye basis and found that target CCFD% measurements did not increase prior to the onset of hyperTDs. We then grouped the target and background boxes from all of the eyes over time and found consistent results.

Our sample, comprised of 27 cases with four longitudinal visits for each case, offers strong evidence on the temporal sequence of RPE/photoreceptor degeneration and decreased CC perfusion. However, we acknowledge the limitations posed by the relatively small sample size and the retrospective nature of the study. Furthermore, cases with extensive pre-existing calcified drusen or HRF overlying regions that later developed hyperTDs were not included due to the inability to visualize the CC in those areas. Despite these limitations, we believe that the consistency of our results shows that increased CCFDs do not precede the onset of hyperTD across all eyes.

Another limitation of our study is our use of a grid strategy that employed a standard grid box size, which may correspond to slightly different physical areas in different eyes due to variations in axial length.^33^ However, this variation would not have impacted our assessments of CCFD% changes performed on individual eyes. Additionally, all eyes had axial lengths under 26 mm, which is within the normal range. Previous reports by Zheng et al.^35^ and Shi et al.^29^ showed no correlation between CCFD area and axial length, as well as no influence of axial length on flow deficit percentages, confirming that the differences in axial length would be unlikely to affect our conclusions. Additional potential confounding factors in our study were the facts that two eyes underwent cataract surgery between visits 1 and 2, and, after the hyperTDs formed, two additional eyes underwent cataract surgery, two eyes started pegcetacoplan therapy, and one eye started anti-VEGF therapy. However, our conclusion that there were no significant changes in the CCFD% measurements before the onset of hyperTDs remains supported by our results.

Finally, the measurement of CCFD% uses a binary metric indicating the absence of detectable flow below a specific signal threshold. This threshold is dependent upon several factors, including but not limited to, the scanning speed and wavelength of the imaging device, the angiographic algorithm, and light-scattering properties of the medium through which the signal passes.^24^^,^^60^ Therefore, we cannot exclude the possibility that the CC perfusion is still present even within flow deficits, but at a velocity below the detectable limits of our SS-OCTA technology. Similarly, we also cannot exclude the possibility that CC velocity may have been reduced prior to the onset of hyperTD but was still detectable using our technology. Therefore, future work will focus on incorporating the dynamic information of CC flow velocity through technologies such as variable interscan time analysis, which has been reported to show a decrease in CC blood flow velocity after the formation of hyperTDs.^72^

Our current results support the proposal that CCFDs do not routinely increase prior to or at the onset of hyperTDs, but decreased CC perfusion develops after the onset of hyperTDs. This suggests a two-stage model for the progression of AMD, with CC involvement and possibly a decrease in choroidal perfusion having more prominent roles in disease progression after the RPE/photoreceptor complex degenerates to form large hyperTDs.

## References

[1] Fleckenstein M, Keenan TDL, Guymer RH, et al. Age-related macular degeneration. *Nat Rev Dis Primers*. 2021; 7(1): 31. 33958600 10.1038/s41572-021-00265-2 PMC12878645

[2] Guymer RH, Rosenfeld PJ, Curcio CA, et al. Incomplete retinal pigment epithelial and outer retinal atrophy in age-related macular degeneration: Classification of Atrophy Meeting Report 4. *Ophthalmology*. 2020; 127(3): 394–409. 31708275 10.1016/j.ophtha.2019.09.035 PMC7218279

[3] Jaffe GJ, Chakravarthy U, Freund KB, et al. Imaging features associated with progression to geographic atrophy in age-related macular degeneration: Classification of Atrophy Meeting Report 5. *Ophthalmol Retina*. 2021; 5(9): 855–867. 33348085 10.1016/j.oret.2020.12.009

[4] Sadda SR, Guymer R, Holz FG, et al. Consensus definition for atrophy associated with age-related macular degeneration on OCT: Classification of Atrophy Report 3. *Ophthalmology*. 2018; 125(4): 537–548. 29103793 10.1016/j.ophtha.2017.09.028 PMC11366072

[5] Lutty G, Grunwald J, Majji AB, Uyama M, Yoneya S. Changes in choriocapillaris and retinal pigment epithelium in age-related macular degeneration. *Mol Vis*. 1999; 5: 35. 10562659

[6] McLeod DS, Grebe R, Bhutto I, Merges C, Baba T, Lutty GA. Relationship between RPE and choriocapillaris in age-related macular degeneration. *Invest Ophthalmol Vis Sci*. 2009; 50(10): 4982–4991. 19357355 10.1167/iovs.09-3639 PMC4829357

[7] Lejoyeux R, Benillouche J, Ong J, et al. Choriocapillaris: fundamentals and advancements. *Prog Retin Eye Res*. 2022; 87: 100997. 34293477 10.1016/j.preteyeres.2021.100997

[8] Faust CD, Klettner CA, Toso M, et al. The vascular geometry of the choriocapillaris is associated with spatially heterogeneous molecular exchange with the outer retina. *J Physiol*. 2024; 602(7): 1273–1295. 38513000 10.1113/JP285050 PMC13547138

[9] Blaauwgeers HG, Holtkamp GM, Rutten H, et al. Polarized vascular endothelial growth factor secretion by human retinal pigment epithelium and localization of vascular endothelial growth factor receptors on the inner choriocapillaris. Evidence for a trophic paracrine relation. *Am J Pathol*. 1999; 155(2): 421–428. 10433935 10.1016/S0002-9440(10)65138-3 PMC1866848

[10] Tezel TH, Geng L, Kaplan HJ, Del Priore LV. Retinal pigment epithelium rescues vascular endothelium from retinoic acid-induced apoptosis. *Invest Ophthalmol Vis Sci*. 2006; 47(11): 5075–5087. 17065529 10.1167/iovs.05-1557

[11] Saint-Geniez M, Kurihara T, Sekiyama E, Maldonado AE, D'Amore PA. An essential role for RPE-derived soluble VEGF in the maintenance of the choriocapillaris. *Proc Natl Acad Sci USA*. 2009; 106(44): 18751–18756. 19841260 10.1073/pnas.0905010106 PMC2774033

[12] Chirco KR, Sohn EH, Stone EM, Tucker BA, Mullins RF. Structural and molecular changes in the aging choroid: implications for age-related macular degeneration. *Eye (Lond)*. 2017; 31(1): 10–25. 27716746 10.1038/eye.2016.216 PMC5233940

[13] Wang S, Li W, Chen M, Cao Y, Lu W, Li X. The retinal pigment epithelium: functions and roles in ocular diseases. *Fundam Res*. 2024; 4(6): 1710–1718. 39734536 10.1016/j.fmre.2023.08.011 PMC11670733

[14] Ochoa Hernandez ME, Lewis-Lujan LM, Burboa Zazueta MG, et al. Role of oxidative stress and inflammation in age related macular degeneration: insights into the retinal pigment epithelium (RPE). *Int J Mol Sci*. 2025; 26(8): 3463. 40331961 10.3390/ijms26083463 PMC12026614

[15] Waheed NK, Moult EM, Fujimoto JG, Rosenfeld PJ. Optical coherence tomography angiography of dry age-related macular degeneration. *Dev Ophthalmol*. 2016; 56: 91–100. 27023214 10.1159/000442784 PMC5875686

[16] Laiginhas R, Shi Y, Shen M, et al. Persistent hypertransmission defects detected on en face swept source optical computed tomography images predict the formation of geographic atrophy in age-related macular degeneration. *Am J Ophthalmol*. 2022; 237: 58–70. 34785169 10.1016/j.ajo.2021.11.001 PMC9035026

[17] Shi Y, Yang J, Feuer W, Gregori G, Rosenfeld PJ. Persistent hypertransmission defects on en face OCT imaging as a stand-alone precursor for the future formation of geographic atrophy. *Ophthalmol Retina*. 2021; 5(12): 1214–1225. 33610834 10.1016/j.oret.2021.02.004

[18] Liu J, Laiginhas R, Corvi F, et al. Diagnosing persistent hypertransmission defects on en face OCT imaging of age-related macular degeneration. *Ophthalmol Retina*. 2022; 6(5): 387–397. 35093585 10.1016/j.oret.2022.01.011 PMC9152950

[19] Liu J, Shen M, Laiginhas R, et al. Onset and progression of persistent choroidal hypertransmission defects in intermediate AMD: a novel clinical trial endpoint: hypertransmission defects as a clinical trial endpoint. *Am J Ophthalmol*. 2023; 254: 11–22. 36958537 10.1016/j.ajo.2023.03.012 PMC10514236

[20] Beqiri S, Herrera G, Liu J, et al. Evaluating the persistence of large choroidal hypertransmission defects using SS-OCT imaging. *Exp Eye Res*. 2024; 248: 110117. 39368694 10.1016/j.exer.2024.110117 PMC11532011

[21] Berni A, Shen M, Cheng Y, et al. The total macular burden of hyperreflective foci and the onset of persistent choroidal hypertransmission defects in intermediate AMD. *Am J Ophthalmol*. 2024; 267: 61–75. 38944135 10.1016/j.ajo.2024.06.023 PMC11486582

[22] Berni A, Kastner JD, Shen M, et al. Hyperreflective foci along the retinal pigment epithelium predict the onset of large choroidal hypertransmission defects in AMD. *Am J Ophthalmol*. 2025; 274: 76–90. 39987980 10.1016/j.ajo.2025.02.021 PMC12492339

[23] El-Mulki OS, Berni A, Kastner J, et al. The macular burden of calcified drusen and the onset of large choroidal hypertransmission defects in intermediate AMD. *Am J Ophthalmol*. 2025; 278: 402–412. 40628339 10.1016/j.ajo.2025.07.001

[24] Chu Z, Zhang Q, Zhou H, et al. Quantifying choriocapillaris flow deficits using global and localized thresholding methods: a correlation study. *Quant Imaging Med Surg*. 2018; 8(11): 1102–1112. 30701164 10.21037/qims.2018.12.09 PMC6328379

[25] Zhang Q, Shi Y, Zhou H, et al. Accurate estimation of choriocapillaris flow deficits beyond normal intercapillary spacing with swept source OCT angiography. *Quant Imaging Med Surg*. 2018; 8(7): 658–666. 30211033 10.21037/qims.2018.08.10 PMC6127524

[26] Zhang Q, Zheng F, Motulsky EH, et al. A novel strategy for quantifying choriocapillaris flow voids using swept-source OCT angiography. *Invest Ophthalmol Vis Sci*. 2018; 59(1): 203–211. 29340648 10.1167/iovs.17-22953 PMC5770182

[27] Chu Z, Gregori G, Rosenfeld PJ, Wang RK. Quantification of choriocapillaris with optical coherence tomography angiography: a comparison study. *Am J Ophthalmol*. 2019; 208: 111–123. 31323202 10.1016/j.ajo.2019.07.003 PMC6889046

[28] Thulliez M, Zhang Q, Shi Y, et al. Correlations between choriocapillaris flow deficits around geographic atrophy and enlargement rates based on swept-source OCT imaging. *Ophthalmol Retina*. 2019; 3(6): 478–488. 31174669 10.1016/j.oret.2019.01.024 PMC11402513

[29] Shi Y, Zhang Q, Zheng F, et al. Correlations between different choriocapillaris flow deficit parameters in normal eyes using swept source OCT angiography. *Am J Ophthalmol*. 2020; 209: 18–26. 31562858 10.1016/j.ajo.2019.09.017 PMC7017580

[30] Chu Z, Zhang Q, Gregori G, Rosenfeld PJ, Wang RK. Guidelines for imaging the choriocapillaris using OCT angiography. *Am J Ophthalmol*. 2021; 222: 92–101. 32891694 10.1016/j.ajo.2020.08.045 PMC7930158

[31] Shen M, Li J, Shi Y, et al. Decreased central macular choriocapillaris perfusion correlates with increased low luminance visual acuity deficits. *Am J Ophthalmol*. 2023; 253: 1–11. 37142175 10.1016/j.ajo.2023.04.011 PMC10626399

[32] Zhou SW, Zhang Y, Noam N, et al. The impact of carotid endarterectomy on choriocapillaris perfusion. *Invest Ophthalmol Vis Sci*. 2023; 64(15): 42. 10.1167/iovs.64.15.42 PMC10756242 38153750

[33] Hiya FE, Cheng Y, Shen M, et al. A novel grid strategy for correlating focal macular anatomic changes with focal changes in choriocapillaris perfusion. *Invest Ophthalmol Vis Sci*. 2024; 65(14): 5. 10.1167/iovs.65.14.5 PMC11620015 39625442

[34] Wang RK, An L, Francis P, Wilson DJ. Depth-resolved imaging of capillary networks in retina and choroid using ultrahigh sensitive optical microangiography. *Opt Lett*. 2010; 35(9): 1467–1469. 20436605 10.1364/OL.35.001467 PMC2864924

[35] Zheng F, Zhang Q, Shi Y, et al. Age-dependent changes in the macular choriocapillaris of normal eyes imaged with swept-source optical coherence tomography angiography. *Am J Ophthalmol*. 2019; 200: 110–122. 30639367 10.1016/j.ajo.2018.12.025 PMC6513331

[36] Shi Y, Zhang Q, Zhou H, et al. Correlations between choriocapillaris and choroidal measurements and the growth of geographic atrophy using swept source OCT imaging. *Am J Ophthalmol*. 2021; 224: 321–331. 33359715 10.1016/j.ajo.2020.12.015 PMC8058170

[37] Heier JS, Lad EM, Holz FG, et al. Pegcetacoplan for the treatment of geographic atrophy secondary to age-related macular degeneration (OAKS and DERBY): two multicentre, randomised, double-masked, sham-controlled, phase 3 trials. *Lancet*. 2023; 402(10411): 1434–1448. 37865470 10.1016/S0140-6736(23)01520-9

[38] Khanani AM, Patel SS, Staurenghi G, et al. Efficacy and safety of avacincaptad pegol in patients with geographic atrophy (GATHER2): 12-month results from a randomised, double-masked, phase 3 trial. *Lancet*. 2023; 402(10411): 1449–1458. 37696275 10.1016/S0140-6736(23)01583-0

[39] Berni A, Cheng Y, Shen M, et al. Updated guidelines for imaging the choriocapillaris in eyes with age-related macular degeneration using swept-source OCT angiography. *Am J Ophthalmol*. 2025; 278: 52–64. 40467021 10.1016/j.ajo.2025.05.021

[40] Laiginhas R, Liu J, Shen M, et al. Multimodal imaging, OCT B-scan localization, and en face OCT detection of macular hyperpigmentation in eyes with intermediate age-related macular degeneration. *Ophthalmol Sci*. 2022; 2(2): 100116. 36249700 10.1016/j.xops.2022.100116 PMC9560648

[41] Wang R, Cheng Y. The impact of scattering tail artifacts on the detection of choriocapillaris flow deficits in OCT imaging. *Invest Ophthalmol Vis Sci*. 2023; 64(9): PP0019.

[42] Cheng Y, Hiya F, Li J, et al. Calcified drusen prevent the detection of underlying choriocapillaris using swept-source optical coherence tomography angiography. *Invest Ophthalmol Vis Sci*. 2024; 65(6): 26. 10.1167/iovs.65.6.26 PMC11185265 38884553

[43] Lang A, Carass A, Jedynak BM, Solomon SD, Calabresi PA, Prince JL. Intensity inhomogeneity correction of SD-OCT data using macular flatspace. *Med Image Anal*. 2018; 43: 85–97. 29040910 10.1016/j.media.2017.09.008 PMC6311386

[44] Zhang Q, Zhang A, Lee CS, et al. Projection artifact removal improves visualization and quantitation of macular neovascularization imaged by optical coherence tomography angiography. *Ophthalmol Retina*. 2017; 1(2): 124–136. 28584883 10.1016/j.oret.2016.08.005 PMC5455345

[45] Zhang DQ, Chen SC. A novel kernelized fuzzy C-means algorithm with application in medical image segmentation. *Artif Intell Med*. 2004; 32(1): 37–50. 15350623 10.1016/j.artmed.2004.01.012

[46] Jia Y, Tan O, Tokayer J, et al. Split-spectrum amplitude-decorrelation angiography with optical coherence tomography. *Opt Express*. 2012; 20(4): 4710–4725. 22418228 10.1364/OE.20.004710 PMC3381646

[47] Lin Z, Hu Y, Lan G, et al. Review of artifacts and related processing in ophthalmic optical coherence tomography angiography (OCTA). *Photonics*. 2025; 12(6): 536.

[48] R Foundation for Statistical Computing. R: a language and environment for statistical computing. Available at: https://www.R-project.org/. Accessed October 14, 2025.

[49] Lenth R, Banfai B, Bolker B, et al. Emmeans: estimated marginal means, aka least-squares means. Available at: https://CRAN.R-project.org/package=emmeans. Accessed October 14, 2025.

[50] Bates DM, Mächler M, Bolker B, Walker S. Fitting linear mixed-effects models using lme4. *J Stat Softw*. 2015; 67(1): 1–48.

[51] Wickham H, Averick M, Bryan J, et al. Welcome to the Tidyverse. *J Open Source Softw*. 2019; 4(43): 1686.

[52] Ramrattan RS, van der Schaft TL, Mooy CM, de Bruijn WC, Mulder PG, de Jong PT. Morphometric analysis of Bruch's membrane, the choriocapillaris, and the choroid in aging. *Invest Ophthalmol Vis Sci*. 1994; 35(6): 2857–2864. 8188481

[53] Curcio CA, Saunders PL, Younger PW, Malek G. Peripapillary chorioretinal atrophy: Bruch's membrane changes and photoreceptor loss. *Ophthalmology*. 2000; 107(2): 334–343. 10690836 10.1016/s0161-6420(99)00037-8

[54] Biesemeier A, Taubitz T, Julien S, Yoeruek E, Schraermeyer U. Choriocapillaris breakdown precedes retinal degeneration in age-related macular degeneration. *Neurobiol Aging*. 2014; 35(11): 2562–2573. 24925811 10.1016/j.neurobiolaging.2014.05.003

[55] Seddon JM, McLeod DS, Bhutto IA, et al. Histopathological insights into choroidal vascular loss in clinically documented cases of age-related macular degeneration. *JAMA Ophthalmol*. 2016; 134(11): 1272–1280. 27657855 10.1001/jamaophthalmol.2016.3519 PMC6014730

[56] Grebe R, Mughal I, Bryden W, et al. Ultrastructural analysis of submacular choriocapillaris and its transport systems in AMD and aged control eyes. *Exp Eye Res*. 2019; 181: 252–262. 30807744 10.1016/j.exer.2019.02.018

[57] Edwards MM, McLeod DS, Shen M, et al. Clinicopathologic findings in three siblings with geographic atrophy. *Invest Ophthalmol Vis Sci*. 2023; 64(3): 2. 10.1167/iovs.64.3.2 PMC9983703 36862121

[58] Lengyel I, Tufail A, Hosaini HA, Luthert P, Bird AC, Jeffery G. Association of drusen deposition with choroidal intercapillary pillars in the aging human eye. *Invest Ophthalmol Vis Sci*. 2004; 45(9): 2886–2892. 15326099 10.1167/iovs.03-1083

[59] Mullins RF, Johnson MN, Faidley EA, Skeie JM, Huang J. Choriocapillaris vascular dropout related to density of drusen in human eyes with early age-related macular degeneration. *Invest Ophthalmol Vis Sci*. 2011; 52(3): 1606–1612. 21398287 10.1167/iovs.10-6476 PMC3101687

[60] Zhou H, Dai Y, Gregori G, et al. Automated morphometric measurement of the retinal pigment epithelium complex and choriocapillaris using swept source OCT. *Biomed Opt Express*. 2020; 11(4): 1834–1850. 32341851 10.1364/BOE.385113 PMC7173887

[61] Spaide RF, Fujimoto JG, Waheed NK, Sadda SR, Staurenghi G. Optical coherence tomography angiography. *Prog Retin Eye Res*. 2018; 64: 1–55. 29229445 10.1016/j.preteyeres.2017.11.003 PMC6404988

[62] Corvi F, Tiosano L, Corradetti G, et al. Choriocapillaris flow deficits as a risk factor for progression of age-related macular degeneration. *Retina*. 2021; 41(4): 686–693. 33009219 10.1097/IAE.0000000000002990

[63] Kar D, Corradetti G, Swain TA, et al. Choriocapillaris impairment is associated with delayed rod-mediated dark adaptation in age-related macular degeneration. *Invest Ophthalmol Vis Sci*. 2023; 64(12): 41. 10.1167/iovs.64.12.41 PMC10540875 37768273

[64] Torrell-Belzach N, Souied EH, Le HM, et al. Comparison of choriocapillaris perfusion between swept-source optical coherence tomography angiography and spectral-domain optical coherence tomography angiography in five different choriocapillaris slabs in patients with intermediate age-related macular degeneration. *Graefes Arch Clin Exp Ophthalmol*. 2025; 263(9): 2495–2504. 40515815 10.1007/s00417-025-06874-x PMC12513984

[65] Braun PX, Mehta N, Gendelman I, et al. Global analysis of macular choriocapillaris perfusion in dry age-related macular degeneration using swept-source optical coherence tomography angiography. *Invest Ophthalmol Vis Sci*. 2019; 60(15): 4985–4990. 31791062 10.1167/iovs.19-27861 PMC6890395

[66] Vujosevic S, Toma C, Villani E, et al. Quantitative choriocapillaris evaluation in intermediate age-related macular degeneration by swept-source optical coherence tomography angiography. *Acta Ophthalmol*. 2019; 97(6): e919–e926. 30900822 10.1111/aos.14088

[67] Zhang A, Zhang Q, Chen CL, Wang RK. Methods and algorithms for optical coherence tomography-based angiography: a review and comparison. *J Biomed Opt*. 2015; 20(10): 100901. 26473588 10.1117/1.JBO.20.10.100901 PMC4881033

[68] Lane M, Moult EM, Novais EA, et al. Visualizing the choriocapillaris under drusen: comparing 1050-nm swept-source versus 840-nm spectral-domain optical coherence tomography angiography. *Invest Ophthalmol Vis Sci*. 2016; 57(9): OCT585–OCT590. 27547891 10.1167/iovs.15-18915 PMC4995042

[69] Zhang Q, Chen CL, Chu Z, et al. Automated quantitation of choroidal neovascularization: a comparison study between spectral-domain and swept-source OCT angiograms. *Invest Ophthalmol Vis Sci*. 2017; 58(3): 1506–1513. 28273317 10.1167/iovs.16-20977 PMC5361585

[70] Ledesma-Gil G, Fernandez-Avellaneda P, Spaide RF. Swept-source optical coherence tomography angiography imaging of the choriocapillaris. *Retina*. 2021; 41(7): 1373–1378. 33411477 10.1097/IAE.0000000000003109

[71] Alagorie AR, Corradetti G, Byon I, et al. Impact of slab selection on the relationship between choriocapillaris flow deficits and enlargement rate of geographic atrophy. *Eye (Lond)*. 2024; 38(5): 847–852. 37865725 10.1038/s41433-023-02788-2 PMC10966059

[72] Hwang Y, Jamil MU, Babiker F, et al. Large hypertransmission defects exhibit greater choriocapillaris flow speed impairment in non-exudative age-related macular degeneration. *Invest Ophthalmol Vis Sci*. 2025; 66(10): PP0016. 10.1016/j.xops.2025.101060 PMC12914210 41717238

